# Supplementary material for: Single-molecule reconstruction of eukaryotic factor-dependent transcription termination
Source: Nat Commun. 2024 Jun 15;15:5113. doi: 10.1038/s41467-024-49527-z (PMC11180205; doi:10.1038/s41467-024-49527-z)
Supplement: Supplementary file 1 — Supplementary Information [file 41467_2024_49527_MOESM1_ESM.pdf]

SUPPLEMENTARY INFORMATION

Single-molecule reconstruction of eukaryotic factor-dependent transcription termination

Xiong et al.

Supplementary Notes

Fitting functions for single-molecule data

Fit the transcription elongation time to single-Gaussian function. The elongation time contains multiple steps of Pol II translocation along DNA substrates (Fig. 1c). Each step of Pol II elongation is a Poisson process that follows a single-exponential function,

$$f(\tau) = -k \cdot exp(-k\tau) \quad (1)$$

where *k* is the rate constant for one-step-forward movement. The total time of Pol II elongation is a convolution of multiple single-exponential functions,

$$f(\tau) = \frac{k^n \tau^{n-1} e^{-k\tau}}{(n-1)!} \quad (2)$$

where n is the number of steps. In the limit of large n, the above function becomes a single-Gaussian function [1]. Therefore, the distribution of Pol II elongation time can be described by a single-Gaussian function.

Fit the termination time to single-molecule Michaelis-Menten function. The termination time is normally composed of the processes of Sen1 binding, which requires Sen1 to search its target in solution and thus relies on Sen1 concentration, and Sen1 catalysis, which possibly requires the formation of Sen1-Pol II TEC intermediate. Sen1 binding process is a Poisson process whose duration follows a single-exponential function. The distributions of the termination time of all Sen1 concentrations display a single-exponential decay suggesting a rate-limiting step for Sen1 binding (Fig. 1e). No fast rise can be observed from the distributions suggesting a fast rate for Sen1 catalysis. Therefore Sen1 binding rate (k_+) can be estimated by fitting the termination time to a single-exponential function,

$$f(\tau) = A \cdot exp(-k_+ S \tau) \quad (3)$$

where S is Sen1 concentration. This gives $k_+ = (5.5 \pm 0.2) \times 10^6$ $M^{-1}s^{-1}$ with a reduced Chi-square of 1.2 (Supplementary Fig. 4a). Considering the effect of Cy3 photobleaching which also follows a single-exponential decay, the Sen1 binding rate (k_+) can be corrected by using the following function,

$$f(\tau) = A \cdot exp(-k_+ S \tau) + B \cdot exp(-\frac{\tau}{t_0}) \quad (4)$$

where t_0 is the mean bleaching time for Cy3 determined in Supplementary Fig. 3a. This gives $k_+ = (6.7 \pm 0.4) \times 10^6$ $M^{-1}s^{-1}$ with a reduced Chi-square of 1.0 (Supplementary Fig. 4b).

Considering the fast step of Sen1 catalysis whose rate is determined by the inverse of the mean duration

of Sen1 HD-SNAP649 presence prior to termination (k_{cat} = 4.04 s^{-1}), the distributions of Sen1 termination time in Fig. 1e can be fit to the single-molecule Michaelis-Menten function corrected with Cy3 photobleaching,

$$f(\tau) = \frac{k_+^0 k_{cat}}{2a}\{exp[(a+b)\tau] - exp[(b-a)\tau]\} + B \cdot exp(-\frac{\tau}{t_0}), \quad (5)$$

where $a = \sqrt{\frac{1}{4}(k_+^0 + k_- + k_{cat})^2 - k_+^0 k_{cat}}$, $b = -\frac{1}{2}(k_+^0 + k_- + k_{cat})$, $k_+^0 = k_+[Sen1\ HD]$. k_{cat} = 4.04 s^{-1} and t_0 = 237 s are held during the fit. The fit gives k_+ = (6.8 ± 13.2) × 10^6 $M^{-1}s^{-1}$ and k_- = (-1.9 ± 7.8) × 10^{-16} s^{-1} with a reduced Chi-square of 1.0. Because k_- is very close to zero, we then constrain k_- as zero and reperform the fit and get k_+ = (6.8 ± 0.4) × 10^6 $M^{-1}s^{-1}$ with a reduced Chi-square of 1.0 (Supplementary Fig. 4c), which is consistent with that estimated with single-exponential function above. These fit results describe that Sen1 diffuses and searches its target at a rate of k_+ prior to the catalytic process.

The catalytic process (k_{cat}) represents the kinetics of Sen1-Pol II TEC intermediate where a single Sen1 is present as observed in the single-molecule fluorescence assays. In this process, Sen1 may not target the stalled Pol II directly because Sen1 can preferentially bind to the RNA transcript of the Pol II TEC. Thus, an RNA-dependent translocation process of Sen1 possibly exists. The distributions of Sen1 residence time prior to termination is then fit to a two-step model.

$$f(\tau) = \frac{k_1 k_2}{k_2 - k_1}[exp(-k_1\tau) - exp(-k_2\tau)] \quad (6)$$

This function is simplified from the above single-molecule Michaelis-Menten function where [Sen1 HD] = 1 representing a single Sen1 molecule and k_- = 0 representing an irreversible reaction. By titrating ATP concentrations, the catalytic process can be separated into an ATP-dependent step (k_1) and an ATP-independent step (k_2). In addition to effect of ATP concentration (Fig. 2c and 3b), the effect of RNA length on k_1 (Supplementary Fig. 8a) as well as the mismatch of transcription bubble on k_2 (Fig. 5c) are examined.

Fit post-termination time to single-exponential function. The kinetics of Sen1 in the post-termination state is characterized by the residence time of Sen1 after RNA release. The distributions of the residence time for all Sen1 concentrations display a single-exponential decay reflecting the dissociation of Sen1 from the RNA, i.e. a Poisson process. The post-termination time of Sen1 is fit to single-exponential function.

Fit to 1D diffusion model. To interpretate the diffusion behavior of Pol II after termination, the lifetime from the termination until the appearance of Cy5 PIFE is measured on Pol II TECs with different distances between

Cy5 and the termination site, 10, 19, 49, 61 and 91 bp respectively, and the mean lifetimes (0.4 ± 0.1 s, 1.0 ± 0.3 s, 1.7 ± 1.4 s, 5.2 ± 4.0 s and 10.4 ± 3.9 s, SEM, respectively) are obtained by averaging individual lifetime events. These data are then fit to 1D diffusion model as previously proposed [2],

$$f(x) = t \cdot \left(1 - \frac{1}{\cosh\left(\frac{x}{L}\right)}\right) \quad (7)$$

where *x* is the distance between Cy5 and the termination site, *t* is the dissociation time, diffusion coefficient equals to L^2/t.

The fraction of Pol II diffusion is calculated the number of Pol II molecules showing Cy5 PIFE over the total number of termination events (112/473, 49/172, 56/552, 14/187, 19/500 and 0/116 for 10, 19, 49, 61, 91 and 539 bp respectively), and is fit to the following function (0/116 not included),

$$f(x) = Frac \cdot \left(1 - \frac{1}{\cosh\left(\frac{x}{L}\right)}\right) \quad (8)$$

where *Frac* is the probability of Pol II diffusion after termination. No Cy5 PIFE data has been obtained on the complex with 539 bp distance and thus not included for the fit.

Fit to 3D diffusion model. The fractions of Pol II diffusion are fit to the below function [3],

$$f(x) = \frac{A}{x}$$

Supplementary Figures

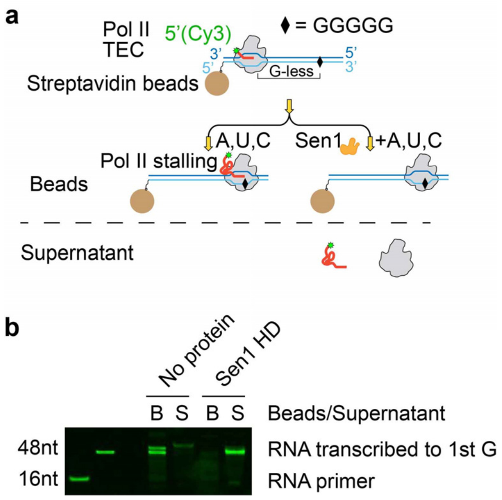

Supplementary Fig. 1 *In vitro* characterization of transcription elongation activity of the reconstructed Pol II TEC and its termination activity mediated by Sen1 HD. (a) Schematic of *in vitro* transcription assay. Reconstructed Pol II TECs are tethered to streptavidin-coated beads allowing, after addition of three nucleotides (A, U and C) without or with Sen1 HD, detection of RNA polymerization remained in beads phase as well as RNA release in supernatant phase. (b) Analysis of RNA generation in beads and supernatant phases in the absence and presence of Sen1 HD. Three experiments are repeated with similar results. Source Date is provided at the end of the Supplementary Information file.

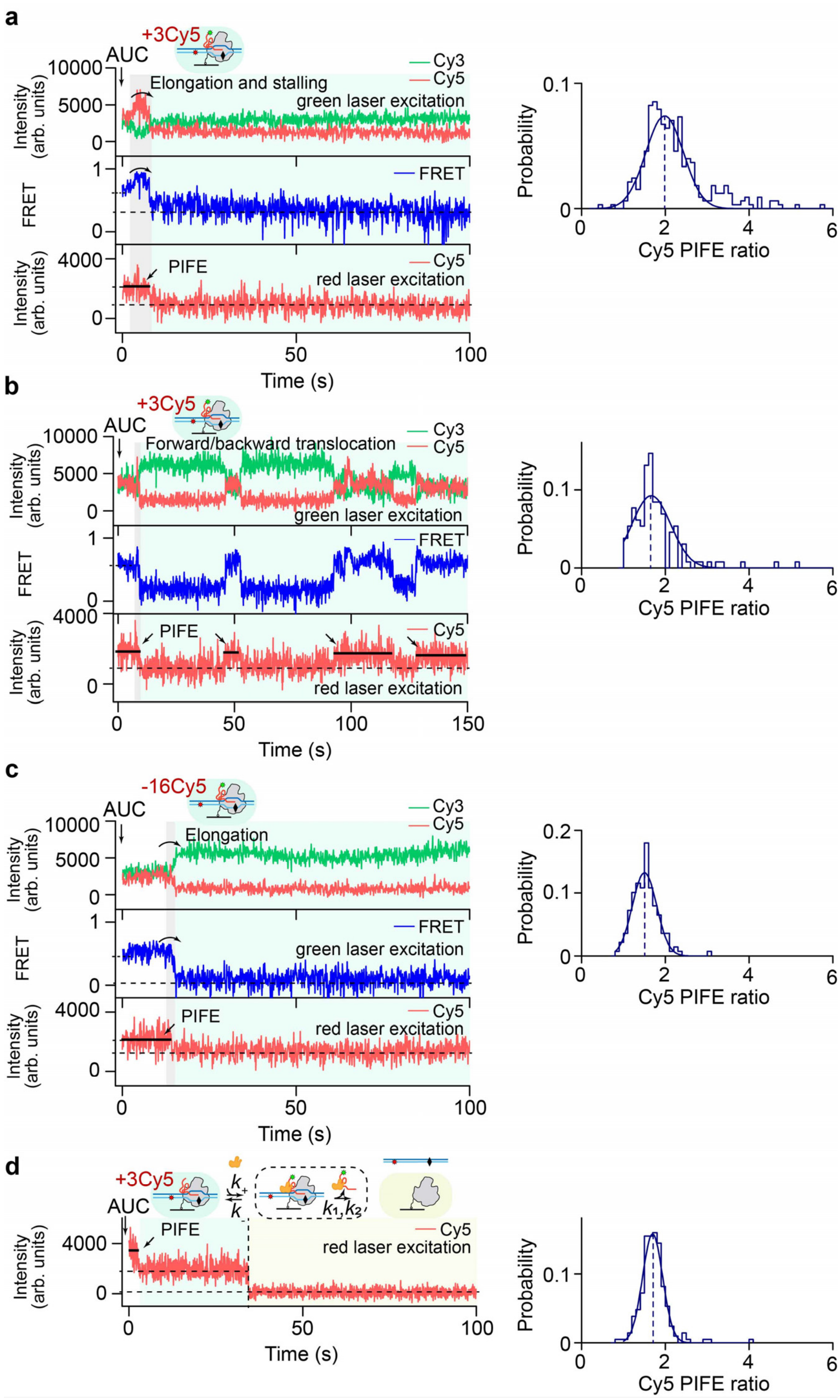

Supplementary Fig. 2 Typical time trajectories for Pol II transcription on different Pol II TECs. (a) Pol II TECs reconstructed with RNA-Cy3 and DNA-Cy5(+3) (bioPol II/RNA-Cy3/DNA-Cy5(+3), Supplementary Data 1)

restarts transcription upon addition of starved nucleotides and TFIIS, reflected by FRET changes from ~0.6 to ~0.8 and then decreasing to ~0.2. The final FRET value stabilizes at ~0.2 for a relatively long time suggesting a stably stalled Pol II at the G-stretch. (b) 112 out of 501 Pol II molecules translocate backward and forward as reflected by FRET fluctuations between ~0.8 and ~0.2, which are coincided with Cy5 PIFE. (c) Pol II TECs with RNA-Cy3 and DNA-Cy5(-16) (bioPol II/RNA-Cy3/DNA-Cy5(-16), Supplementary Data 1) restarts transcription showing direct decreases in FRET (~0.5 to ~0) and Cy5 PIFE. (d) Pol II TECs reconstructed the same as that in (a) but without Cy3 labeling showing Cy5 PIFE prior to DNA-Cy5 dissociation caused by Sen1-dependent termination. Histogram of Cy5 PIFE ratio are each fit to single-Gaussian function yielding peaks at 1.98 ± 0.03, 1.66 ± 0.06, 1.50 ± 0.03, and 1.70 ± 0.02 (SEM, N = 328, 130, 139 and 208, respectively). The number of backtracking events is larger than that of backtracking trajectories because Pol II backtracks more than once on several trajectories. Source data are provided as a Source Data file.

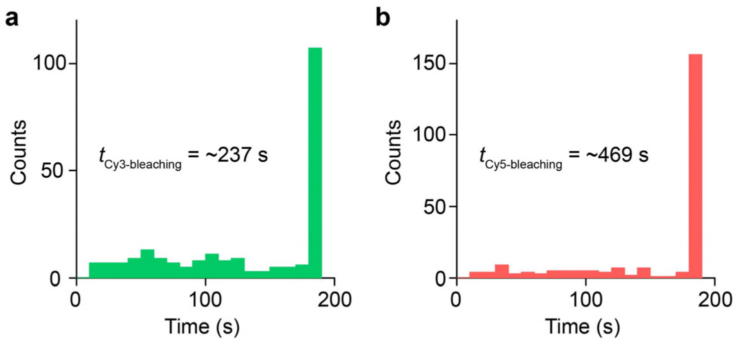

Supplementary Fig. 3 Duration histograms of Cy3 (a) and Cy5 (b) photobleaching due to laser excitation, measured on bioPol II/RNA-Cy3/DNA-Cy5(+3) TECs in imaging buffer under green (8 mW) and red (5 mW) laser excitations for Cy3 and Cy5, respectively. As a fluorophore bleaching follows a single-exponential decay, the fraction of Cy3 or Cy5 that doesn't bleach in the record (180 s) can be used to estimate their mean bleaching time, ~237 s for Cy3 and ~469 for Cy5, N = 229 events. Source data are provided as a Source Data file.

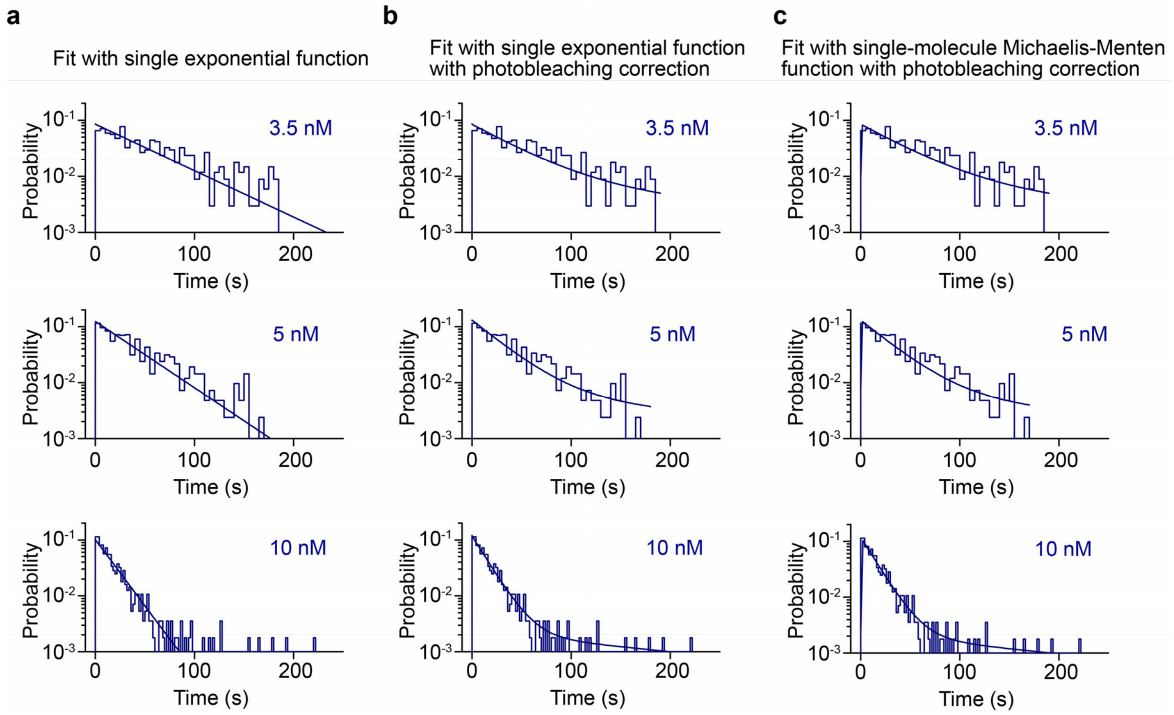

Supplementary Fig. 4 Duration histograms of Pol II termination from Fig. 1d are fit in different ways. (a) Global fit to a single-exponential function giving k_+ = (5.5 ± 0.2) × 10^6 $M^{-1}s^{-1}$ (SEM) with a reduced Chi-square of 1.2. (b) Global fit to a single exponential function with photobleaching correction (the mean photobleaching time for Cy3 is constrained as 237 s) giving k_+ = (6.7 ± 0.4) × 10^6 $M^{-1}s^{-1}$ (SEM) with a reduced Chi-square of 1.0. (c) Global fit to a single-molecule Michaelis-Menten function with photobleaching correction giving k_+ = (6.8 ± 0.4) × 10^6 $M^{-1}s^{-1}$ (SEM) with a reduced Chi-square of 1.0. N = 337, 417 and 564 for 3.5, 5 and 10 nM Sen1 HD concentrations, respectively. See fit details in Methods.

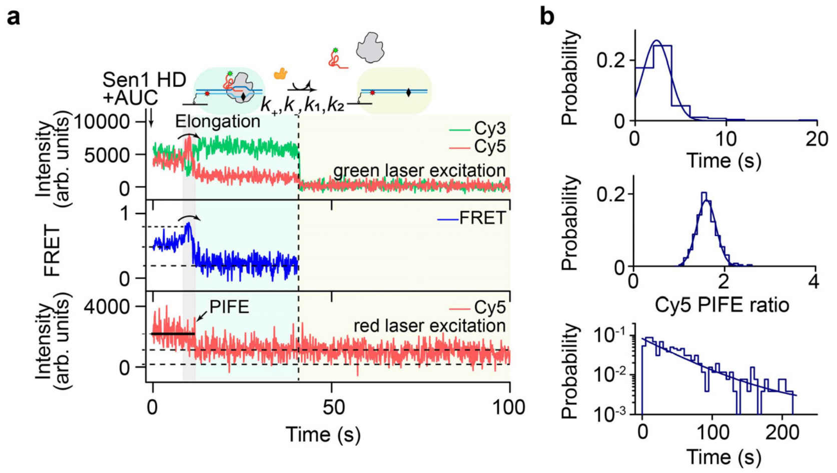

Supplementary Fig. 5 Characterization of Sen1-dependent termination via Pol II TECS with biotinylated DNA. (a) Typical trajectories for Pol II/RNA-Cy3/bioDNA-Cy5(+3) TECs tethered through biotinylated DNA to PEG surface, characterized by FRET changes and Cy5 PIFE (light grey zone), followed by a stable FRET signal at ~0.2 (green zone) and then Cy3 disappearance (dashed line). Cy5 fluorescence lasts for a relatively long time after Cy3 disappearance (i.e., transcription termination). (b) Histograms of the Pol II elongation time (grey zone) give a Gaussian peak at 2.4 ± 0.1 s (SEM, N = 260, upper panel), and the Cy5 PIFE ratio representing a Gaussian peak at 1.60 ± 0.01 (middle panel), and the termination time (green zone) fitting to a single exponential function with photobleaching correction give $k_+ = (4.2 \pm 0.1) \times 10^6\ M^{-1}s^{-1}$ (SEM) with a reduced Chi-square of 0.88 (lower panel). Source data are provided as a Source Data file.

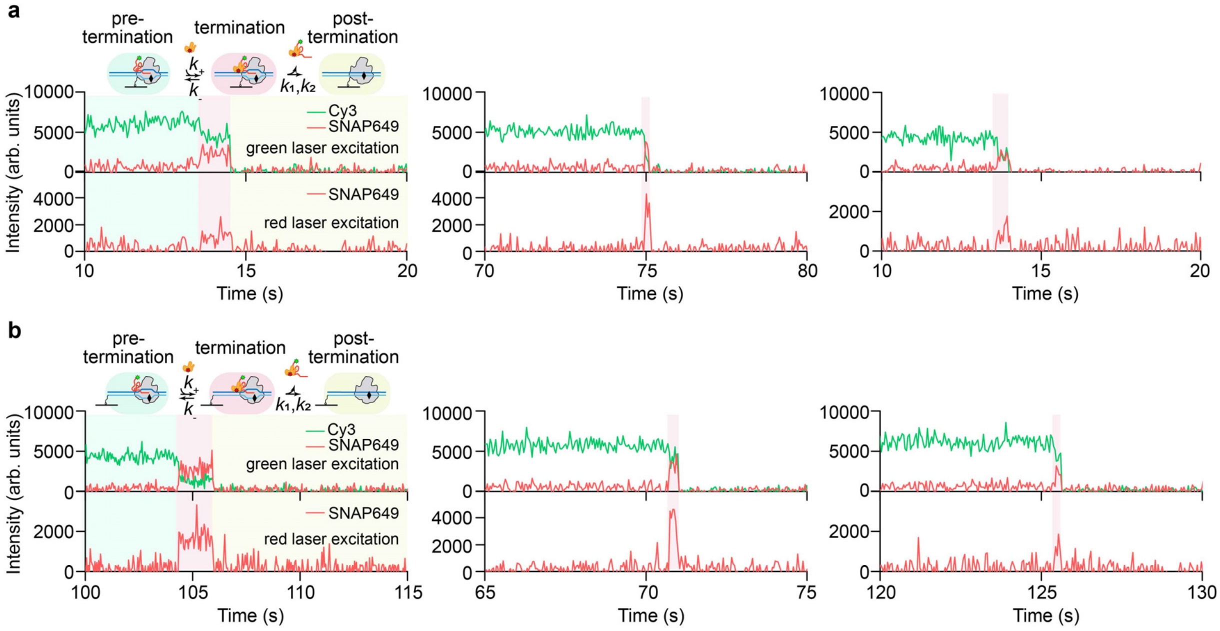

Supplementary Fig. 6 Typical trajectories for Pol II termination by Sen1 HD-SNAP649. (a) Pol II TECs tethered through biotinylated Pol II undergo Sen1 HD-SNAP649 mediated termination showing coexistence of Sen1 HD-SNAP649 and RNA, i.e. Sen1-Pol II TEC complex, prior to their dissociation. A FRET change coincides with the appearance of Sen1 HD-SNAP649 fluorescence (pink zone). (b) Pol II TECs tethered through biotinylated DNA also show the formation of Sen1-Pol II TEC complex reflected by Cy3/Sen1 HD-SNAP649 FRET, and the simultaneous releases of Sen1 HD-SNAP649 and RNA.

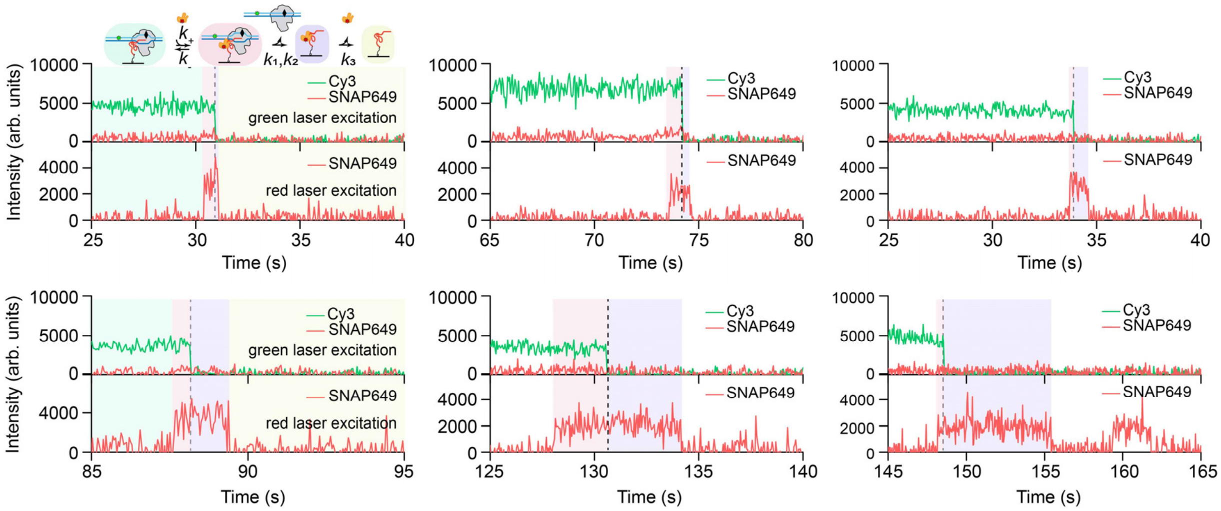

Supplementary Fig. 7 Typical trajectories representing transcription termination on Pol II/bioRNA/DNA-Cy3 TECs. Pol II TECs are tethered to PEG surface through biotinylated RNA, allowing detection of Sen1 HD-SNAP649 action on RNA in the post-termination state. Four zones are identified: a green zone, representing a pre-termination state prior to the appearance of Sen1 HD-SNAP649 fluorescence; a pink zone, representing the formation of Sen1-Pol II TEC intermediate, reflected by the coexistence of Sen1 HD-SNAP649 and RNA-Cy3 prior to termination; a purple zone, representing the post-termination state that Sen1 HD-SNAP649 interacts with RNA, reflected by the lagged disappearance of Sen1 HD-SNAP649 relative to Cy3 disappearance; and a yellow zone with Sen1 HD-SNAP649 having disappeared.

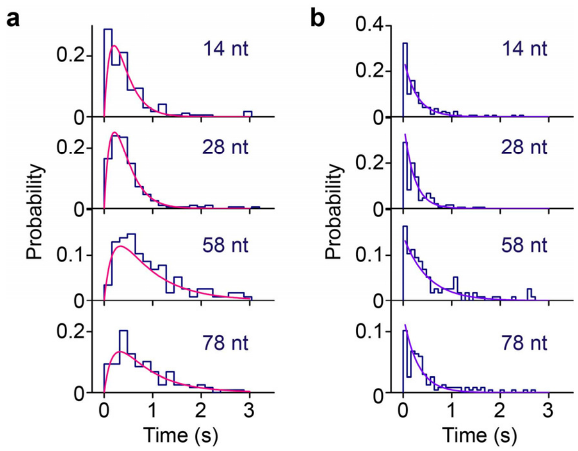

Supplementary Fig. 8 Characterization of Sen1 action during termination and post termination states. (a) Duration histograms of Sen1 HD-SNAP649 action during Sen1-dependent termination at various RNA lengths (14, 28, 58 and 78 nt) that extend from the RNA exit channel of Pol II. Fitting these histograms to single-molecule Michaelis-Menten model gives k_1 = 4.32 ± 1.07, 3.97 ± 0.86, 1.34 ± 0.22, and 1.46 ± 0.24 s^{-1} (SEM, N = 170, 195, 116 and 118 for 14, 28, 58 and 78 nt, respectively) and k_2 = 5.55 ± 1.48 s^{-1} (SEM) with a reduced Chi-square of 1.1. (b) Duration histograms of Sen1 HD-SNAP649 in post-termination state at various RNA lengths. By fitting to single-exponential function, the mean durations are obtained as 3.72 ± 0.60, 4.67 ± 0.61, 1.99 ± 0.30 and 3.35 ± 0.57 s^{-1} (SEM, N = 170, 195, 116 and 118 for 14, 28, 58 and 78 nt, respectively). Source data are provided as a Source Data file.

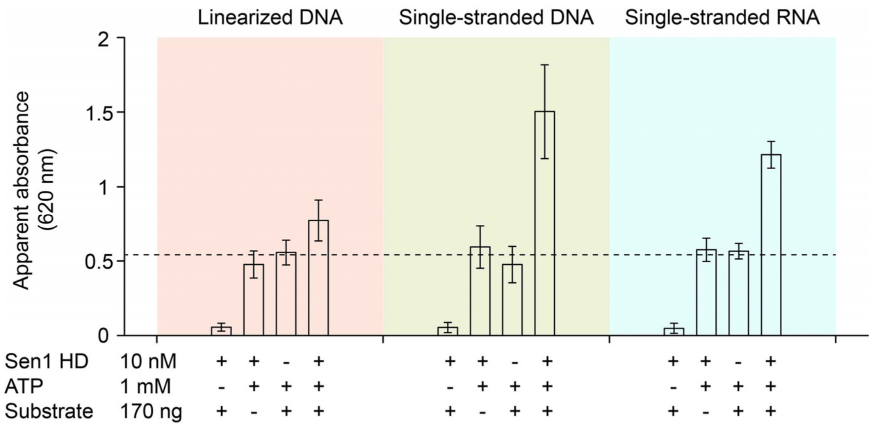

Supplementary Fig. 9 ATPase activity of Sen1 HD on different substrates. This experiment is performed using an ATPase/GTPase activity kit to measure the apparent absorbance at 620 nm of the stable dark green color product formed with free phosphate (mean ± SD, see Methods). Three types of substrates are used, linearized DNA obtained by digesting pUC18 plasmid with KpnI enzyme, 58 nt single-stranded DNA (14nt-Tem, Supplementary Data 3) and 60 nt single-stranded RNA (60mer-RNA, Supplementary Data 3). Control experiments are performed as listed and a background of ~0.6 is observed when ATP is present (dash line). Weak ATPase activity of Sen1 HD on the linearized DNA is observed, which is far lower than that on the single-stranded DNA or RNA. Each experiment is repeated three times with similar results. Source data are provided as a Source Data file.

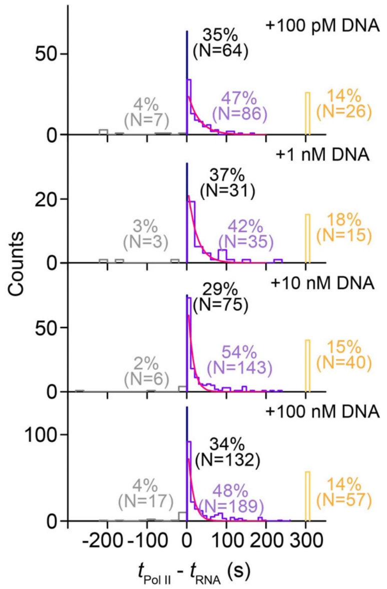

Supplementary Fig. 10 Histograms of the time elapsed between RNA release and Pol II dissociation in the presence of excess DNAs that have been used in Fig. 4a but without Cy5 labeling, grey for Pol II dissociation from DNA before RNA release (possibly because of photobleaching), navy for simultaneous dissociation of Pol II/RNA from DNA (i.e. with no more than one frame of lag at a 10 Hz collection rate), purple for Pol II lag dissociation, and orange for Pol II retaining on DNA over the course of the recording. The mean times of purple events are 28 ± 5 s, 22 ± 5 s, 12 ± 2 s, and 13 ± 2 s (SEM) for 100 pM, 1 nM, 10 nM , and 100 nM DNA, respectively. Source data are provided as a Source Data file.

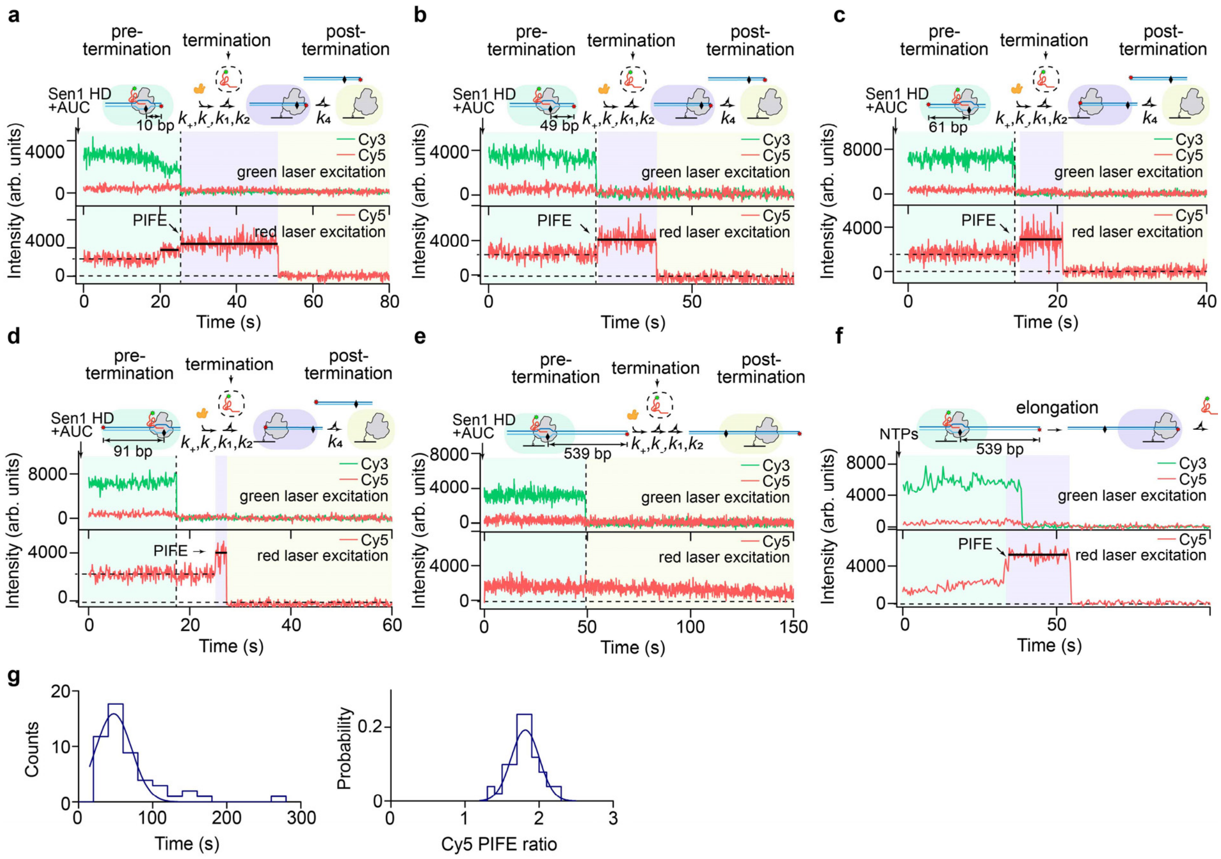

Supplementary Fig. 11 Typical trajectories of Sen1-dependent termination when positioning Cy5 at downstream or upstream DNA ends to generate different distances (a-e). Cy5 PIFE are observed and the lifetimes after RNA release but prior to Cy5 PIFE appearance are measured (a-d). The mean values are presented in Fig. 4g for 1D diffusion fit. (e) No Cy5 PIFE can be observed out of 116 termination events on the bioPol II/RNA-Cy3/DNA539-Cy5(3') complex. (f) Typical trajectories showing the appearance of Cy5 PIFE reflecting Pol II elongation to the downstream end upon addition of NTPs (lower panel) and RNA release (upper panel). (g) Histograms of the lifetime for the appearance of Cy5 PIFE from NTPs injection yielding a Gaussian peak at 47 ± 24 s (SD, N = 51, left panel), and the corresponded Cy5 PIFE ratio at a peak of 1.8 ± 0.03 (SEM, right panel). The fraction of Pol II restarting elongation is 51/104. Source data are provided as a Source Data file.

Supplementary References

1. Xie, S. Single-molecule approach to enzymology. *Single Mol* **2**, 229–236 (2001).

2. Kang, W. *et al.* Transcription reinitiation by recycling RNA polymerase that diffuses on DNA after releasing terminated RNA. *Nat Commun* **11** (2020).

3. Berg, H. C. *Random Walks in Biology* (Princeton Univ. Press, New Jersey, 1983).

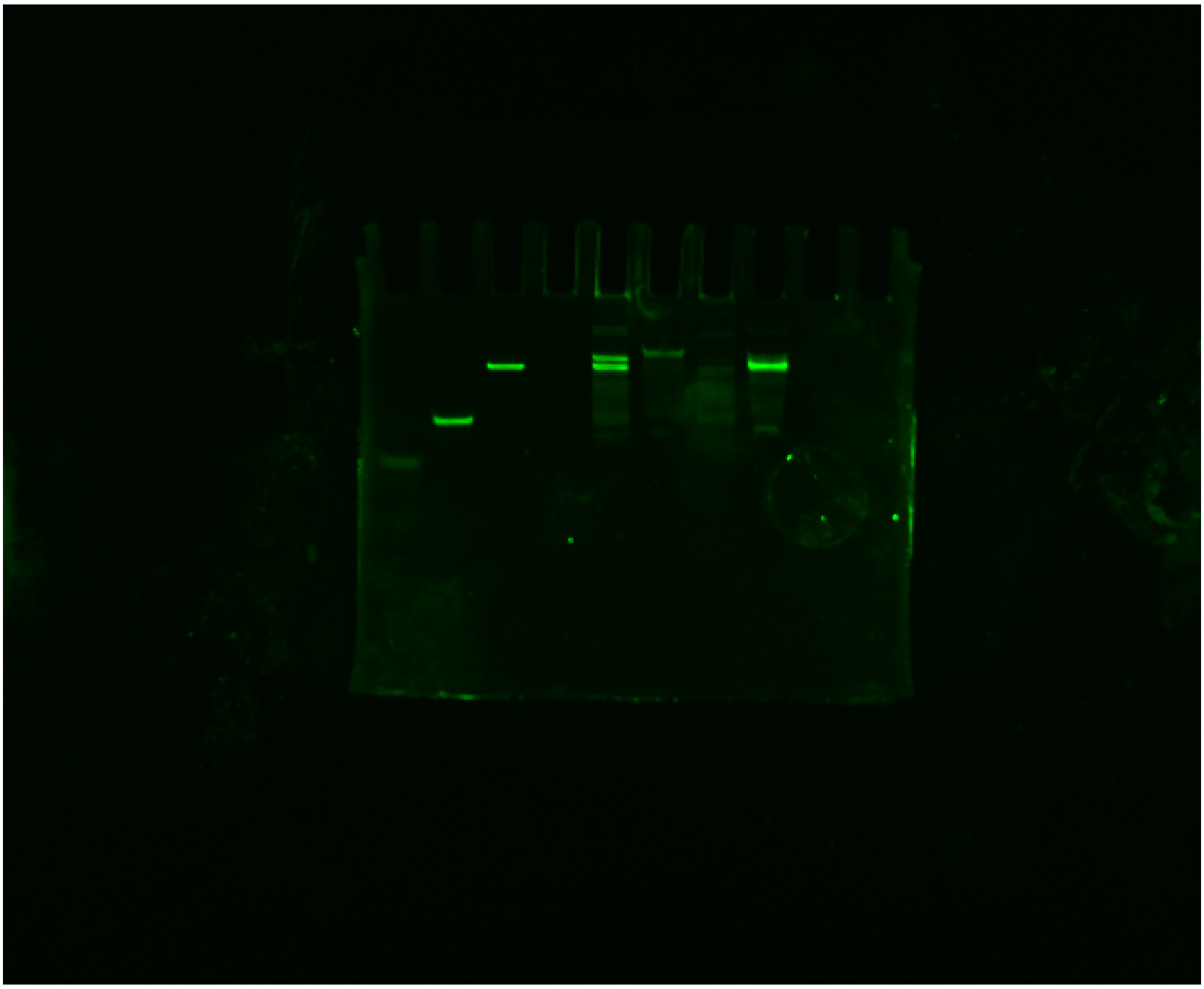

Source Data for Supplementary Fig. 1b.
