# Supplementary material for: Single-molecule reconstruction of eukaryotic factor-dependent transcription termination
Source: Nat Commun. 2024 Jun 15;15:5113. doi: 10.1038/s41467-024-49527-z (PMC11180205; doi:10.1038/s41467-024-49527-z)
Supplement: Supplementary file 3 — Description of Additional Supplementary Files [file 41467_2024_49527_MOESM3_ESM.pdf]

Description of Additional Supplementary Files

File Name: Supplementary Data 1
Description: Labeling strategies for Pol II TECs and Sen1 HD and their applications

File Name: Supplementary Data 2
Description: Summary of fit parameters

File Name: Supplementary Data 3
Description: Oligos used in this study
